# Visualizing the Heterogeneity of Effects in the Analysis of Associations of Multiple Myeloma with Glyphosate Use. Comments on Sorahan, T. Multiple Myeloma and Glyphosate Use: A Re-Analysis of US Agricultural Health Study (AHS) Data. *Int. J. Environ. Res. Public Health* 2015, *12*, 1548–1559

**DOI:** 10.3390/ijerph14010005

**Published:** 2016-12-22

**Authors:** Igor Burstyn, Anneclaire J. De Roos

**Affiliations:** Department of Environmental and Occupational Health, Dornsife School of Public Health, Drexel University, Philadelphia, PA 19104, USA; ajd335@drexel.edu

**Keywords:** simulation, epidemiology, homogeneity, effect size

## Abstract

We address a methodological issue of the evaluation of the difference in effects in epidemiological studies that may arise, for example, from stratum-specific analyses or differences in analytical decisions during data analysis. We propose a new simulation-based method to quantify the plausible extent of such heterogeneity, rather than testing a hypothesis about its existence. We examine the contribution of the method to the debate surrounding risk of multiple myeloma and glyphosate use and propose that its application contributes to a more balanced weighting of evidence.

## 1. Introduction

We wish to comment on the work of Sorahan [1] published in this journal, which influenced an interpretation of De Roos et al. [2] by Acquavella et al. [3]. Specifically, Sorahan [1] asserted that De Roos et al. [2] produced upwardly biased estimates of the association between glyphosate in agriculture and the risk of multiple myeloma. The support for this conclusion rests on a re-analysis of the same cohort. However, we are not convinced that Prof. Sorahan’s results warrant such an assertion. We first present the method we used to evaluate the heterogeneity of the effects and then return to a more in-depth look at the specific claim of Acquavella et al. [3].

## 2. Description of a New Method

It is almost inevitable that an epidemiologist (and scientists using epidemiologic results) will encounter two effect estimates for ostensibly the same exposure–outcome association that look dissimilar. Kaufman and MacLehose [4] demonstrate how to tackle this problem by means of familiar statistical tests (*p*-Values and estimates of difference in effect estimates). We venture to propose the visualization of the important aspect of heterogeneity of effects not captured by such tests. Furthermore, all the usual tests of heterogeneity assume independence of effect estimates; if this is not so, the tests unjustifiably favor a conclusion of homogeneity. In practice, we cannot always justify the assumption of independence, such as when we consider the heterogeneity of effects due to analytical decisions made in the analysis of the same study. Left with no appropriate formal tools, scientists rely on their intuition and its perils.

**Figure 1:**
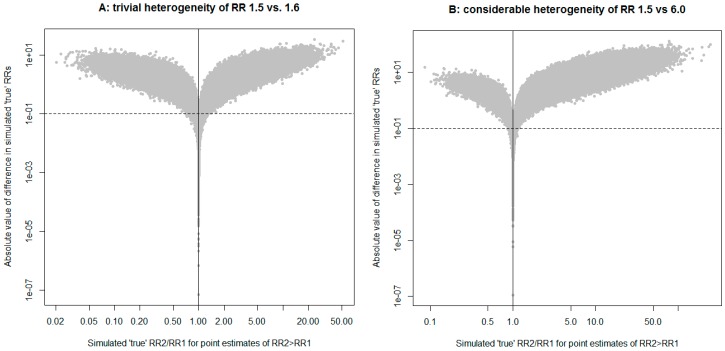
Results of 1,000,000 simulated true rate ratios (RR). When simulated RR are in the same order as point estimates, they appear to the right of solid vertical line; when absolute different in simulated RR is ≥0.1, they appear above dashed horizontal line. (**A**) shows a synthetic example that illustrates virtual homogeneity; (**B**) shows a synthetic example that illustrates considerable heterogeneity. Precision is the same in both panels for higher vs. lower point estimate of RR.

We propose a new procedure that may prove helpful in such a situation (implementation in R [5] is given in Appendix A). Let us assume that the estimates of the rate ratios (RRs) and their variances are unbiased for the sampled population (internally valid); let us also not make the (strong) assertion of homogeneity (i.e., it is a truism in epidemiology that true effects will vary by populations because of myriad uncontrollable factors among populations). We can then simulate true values of RRs that are consistent with the reported estimates by sampling from normal distributions centered on observed log(RR) with observed var(log(RR)). Given two simulated true RRs, we can compare their rank order and absolute differences. The rank order allows us to evaluate how likely it is for the population with the smaller point estimate to have the larger true RR. We also record how frequently the two simulated true RRs are materially different, defined, for example, as differing by ≥±0.1 units of the RR. This procedure does not require any assumption about the independence of the two estimates of the RR, because we assume that the simulated true RRs are independent (i.e., observed values can have some dependence but not underlying true values). Figure 1A,B illustrate the properties of the procedure for synthetic cases of trivial and considerable heterogeneity, respectively. The concentration of the simulation results in the upper right-hand corner of the plot we developed suggests tangible heterogeneity (Figure 1B). We support the plea of Kaufman and MacLehose [4] that unaided eyeballing of these matters is harmful, and yet still we believe in providing scientists with intuitive tools to visualize heterogeneity that matters to them. We hope that our modest contribution to this endeavor will help us all see things more clearly.

## 3. Application to a Case Study of the Risk of Multiple Myeloma in Relation to Glyphosate Use

**Figure 2:**
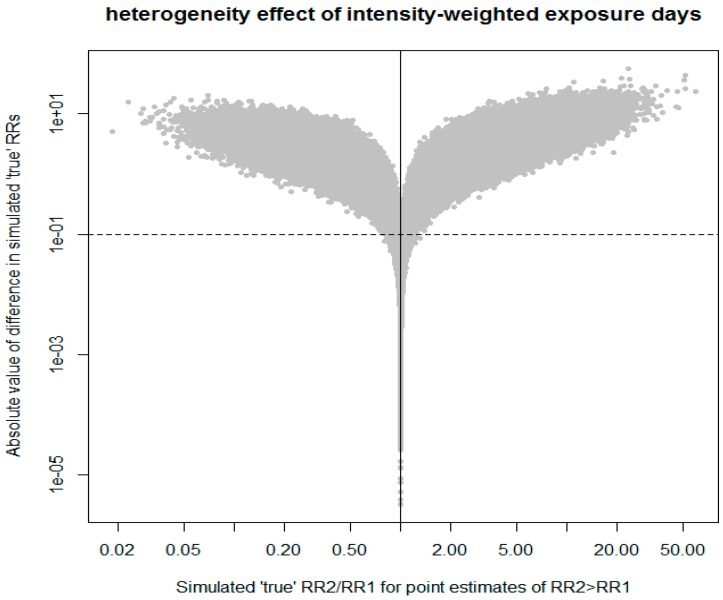
Comparing the results from De Roos et al. [2] with those from Sorahan [1] of the effect estimates for the highest category of intensity-weighted exposure days vs. the reference. When the simulated RRs are in the same order as the point estimates, they appear to the right of the solid vertical line; results of 1,000,000 simulated true RRs (see text for the details).

De Roos et al. [2] reported associations between glyphosate use and the risk of multiple myeloma in the Agricultural Health Study cohort. They contrasted risk for cohort members in the highest category of intensity-weighted exposure days (8 cases) to low-exposed subjects (5 cases) in a Poisson adjusted model that accounted for the use of multiple pesticides, age, gender, and lifestyle factors (30,613 subjects). Sorahan [1] reanalyzed the data and obtained the most comparable estimate to that of De Roos et al. [2] by using the same model, except that (a) the reference group was never-exposed; and (b) subjects with missing values for covariates were included. Inclusion of subjects with some missing values was a strategy to reduce suspected selection bias; the contrast thereby included 10 cases in the highly exposed group vs. 8 cases among never-exposed subjects (54,315 subjects). De Roos et al. [2] reported a RR of 2.1 and a 95% confidence interval (CI) of 0.6–7.0, whereas Sorahan [1] observed a RR of 1.87 and a 95% CI of 0.67–5.27. The analytical choices made by Sorahan [1] produced a lower RR, but is the difference between the two RRs of any consequence? A test of heterogeneity [4] yields a *Z*-statistic of 0.14 and an estimate of the ratio of the two RRs is 1.1 with a 95% CI of 0.2–5.6. If the two estimates were independent, this would provide support for a lack of heterogeneity, but the 95% CI of the ratio of RR is so wide that it admits a multitude of plausible interpretations. The application of our procedure, illustrated in Figure 2, argues against any important heterogeneity. Specifically, the simulation suggests that there is only a 44% chance that the true value of De Roos et al. [2] is actually greater than that of Sorahan [1], contrary to the ranking indicated by the point estimates. This observation renders the evaluation of the difference between the two assumed true RRs to be a moot point (however, we note for completeness that the difference was less than 0.1 (i.e., trivial) in only 5% of the simulations). The simulation leads us to conclude that the work of Sorahan [1] did not uncover any important methodological problem in the analysis of De Roos et al. [2] with respect to the exclusion of subjects with missing data from this exposure–response analysis. In fact, it appears to us that it weakened the evidence for selection bias, playing an important role in the associations with intensity-weighted exposure days. Nonetheless, the later review paper by Acquavella et al. [3] (including Prof. Sorahan among the authors) stresses selection bias in downplaying the contribution of De Roos et al. [2]. They appear to base this on the discrepancy in De Roos et al. [2] for the contrast between ever vs. never exposed subjects obtained from two different selections of cohort members, which is, due to the crudeness of the exposure metric, one of the least informative analyses for the assessment of causality. There is no reason to suppose *a priori* that selection bias, if present, will have the same effect on all exposure metrics. Whereas there is likely selection bias adversely affecting the analysis with ever vs. never exposed subjects following the exclusion of subjects with missing data, there is no evidence that this applies to the more informative exposure–response associations.

## 4. Conclusions

We contend that the work by Sorahan [1] reinforced rather than detracted from the key results of De Roos et al. [2]. In the case of the exposure–response analysis in particular, there is evidence of robustness to the analytical treatment of the data that bolsters the usefulness of the results in risk analysis. There is no evidence of heterogeneity in the risk estimates produced in the two analyses for the intensity-weighted exposure days. We conclude that Acquavella et al. [3] gave undue weight to the re-analysis of De Roos et al. [2] by Sorahan [1] in drawing their conclusions, by focusing on the crude comparison of ever vs. never exposed, rather than the more informative exposure metrics indicating cumulative use. Although the risk estimates for multiple myeloma and glyphosate use of De Roos et al. [2] are certainly imprecise, this does not mean they are uninformative (there is general agreement that they are the best available estimates). We do not take any particular view on the weight of evidence for or against glyphosate use causing multiple myeloma, but are simply concerned with the use of the most informative risk estimates and the better use of information on heterogeneity (or material lack thereof) in weighing evidence. One can only hope that updates of the Agricultural Health Study cohort (or other attempts to replicate the result) will help achieve greater clarity regarding the role of glyphosate use in the risk of multiple myeloma.

## Appendix A

R code to produce evaluation of heterogeneity in the case of intensity-weighted exposure days
#INPUTS INTO SIMULATION
set.seed(585310)
#Sorahan T “the highest category ofintensity-weighted exposure days in the fully adjusted model”
#RR 1.87, 95% CI 0.67 to 5.27
logrr1=log(1.87) #log-RR1
se11=(log(5.27)-log(0.67))/3.92 #log-SE(RR1) estimated from 95% CI

#De Roos highest “Intensity-weighted exposure days” (Table 3 of De Roos et al., 2005)
#RR 2.1, 95% CI 0.6 to 7.0
logrr2=log(2.1)
se22=(log(7)-log(0.6))/3.92

#SIMULATION
#draw samples from each estimate to simulate fix true value of RR
#compare if two estimates are consistent with different true values
#if we take point estimates to be true values than RR1<RR2

n=1000000
true1=rnorm(n,logrr1,se11)
true2=rnorm(n,logrr2,se22)

#chance that RR1 is actually bigger than RR2
SmallBigger <- ifelse(true1>true2,1,0)
100*sum(SmallBigger)/n

#count virtually identical estimates: differ in 2nd digit of RR.
trivial=0.1
rr1<-exp(true1)
rr2<-exp(true2)
ratio<-rr2/rr1
diff2<-abs(rr1-rr2)
same<-ifelse(diff2<trivial, 1,0)
100*sum(same)/n

#plots
#FIGURE2A
plot(ratio, diff2, log=“xy”,
xlab=“Simulated ‘true’ RR2/RR1 for point estimates of RR2>RR1”,
ylab=“Absolute value of difference in simulated ‘true’ RRs”, cex=1, pch=20, col=“#C0C0C0”)
abline(v=1, lty=1)
abline(h =trivial, lty=2)
title(main=“heterogeneity effect of intensity-weighted exposure days”)

#order correct and difference >=trivial
right<-ifelse(SmallBigger==0 & same==0,1,0)
100*sum(right)/n
